# Next-Generation Sequencing Reveals Four Novel Viruses Associated with Calf Diarrhea

**DOI:** 10.3390/v13101907

**Published:** 2021-09-23

**Authors:** Qi Wu, Jizong Li, Wei Wang, Jinzhu Zhou, Dandan Wang, Baochao Fan, Xuehan Zhang, Dongbo Sun, Ga Gong, Sizhu Suolang, Bin Li

**Affiliations:** 1 Institute of Veterinary Medicine, Jiangsu Academy of Agricultural Sciences, Key Laboratory of Veterinary Biological Engineering and Technology Ministry of Agriculture, Nanjing 210014, China; wuqi1109323413@163.com (Q.W.); lijizong22@sina.com (J.L.); 13952032293@126.com (W.W.); syzhoujz@163.com (J.Z.); wddan26@163.com (D.W.); fanbaochao.0405@163.com (B.F.); liuxuehan1996@hotmail.com (X.Z.); 2 Animal Science College, Tibet Agriculture and Animal Husbandry University, Nyingchi 860000, China; 3 Jiangsu Key Laboratory for Food Quality and Safety-State Key Laboratory Cultivation Base of Ministry of Science and Technology, Nanjing 210014, China; 4 Jiangsu Co-Infection Center for Prevention and Control of Important Animal Infectious Disease and Zoonoses, Yangzhou 225009, China; 5 Jiangsu Key Laboratory of Zoonoses, Yangzhou University, Yangzhou 225009, China; 6 Laboratory for the Prevention and Control of Swine Infectious Diseases, College of Animal Science and Veterinary Medicine, Heilongjiang Bayi Agricultural University, Daqing 163319, China; dongbosun@126.com

**Keywords:** next-generation sequencing, genome analysis, calf diarrhea, bovine kobuvirus, bovine norovirus, bovine astrovirus, bovine coronavirus

## Abstract

Calf diarrhea is one of the common diseases involved in the process of calf feeding. In this study, a sample of calf diarrhea that tested positive for bovine coronavirus and bovine astrovirus was subjected to high-throughput sequencing. The reassembly revealed the complete genomes of bovine norovirus, bovine astrovirus, bovine kobuvirus, and the S gene of bovine coronavirus. Phylogenetic analysis showed that the ORF2 region of bovine astrovirus had the lowest similarity with other strains and gathered in the Mamastrovirus unclassified genogroup, suggesting a new serotype/genotype could appear. Compared with the most closely related strain, there are six amino acid mutation sites in the S gene of bovine coronavirus, most of which are located in the S1 subunit region. The bovine norovirus identified in our study was BNoV-GIII 2, based on the VP1 sequences. The bovine kobuvirus is distributed in the Aichi virus B genus; the P1 gene shows as highly variable, while the 3D gene is highly conserved. These findings enriched our knowledge of the viruses in the role of calf diarrhea, and help to develop an effective strategy for disease prevention and control.

## 1. Introduction

Calf diarrhea is a common gastrointestinal disease in calves, and most cases occur at less than 1 month of age. Calf diarrhea often occurs due to environmental contamination with pathogens, and it is transmitted via fecaoral transmission routes. The main clinical features include diarrhea, dehydration, and foul-smelling feces. Morbidity and mortality are high, and the incidence of calf diarrhea was higher when cattle were raised in groups [1]. Prior studies have implicated several viruses in calf diarrhea, and rotavirus is the main viral causative agent of diarrhea in calves worldwide [2]. Moreover, several other viruses, including bovine viral diarrhea virus (BVDV), bovine adenovirus (BAdV), bovine torovirus (BToV), bovine parvovirus (BPV), bovine coronavirus (BCoV), bovine astrovirus (BoAstV), bovine kobuvirus (BKoV), and bovine norovirus (BNoV), have been recognized as the causative agents of diarrhea [3,4,5,6,7]. The economic impact caused by this condition is significant, although many new intervention strategies, such as vaccines, medications, and herd management, have been developed and implemented to minimize economic losses [8].

A metagenomic approach was used to detect all potential pathogens in a sample, this method has great potential utility in the diagnosis of infectious disease [9,10]. The traditional diagnostic techniques in laboratories include viral culture and the molecular identification of viral nucleic acids, most commonly via PCR/RT-PCR. Most molecular assays target only a limited number of pathogens using specific primers or probes, while metagenomic approaches characterize all DNA or RNA present in a sample, enabling the analysis of an entire genome. The metagenomic approach for clinical applications derives its roots from the use of microarrays in the early 2000s [11,12]; these methods have been widely used to identify infections in ancient remains [13], characterize the animal virome in both healthy and diseased states [14,15], and discover novel viral pathogens [16].

In February 2021, calf diarrhea of unknown pathogen broke out on a cattle farm in Shijiazhuang, Hebei province, China. We collected 63 cases of diarrheal calf stool and sent it to the laboratory for pathogen detection. Several diagnostic methods were performed, including the RT-PCR assay, to detect BVDV, BCoV, BoAstV, and BRoV. The results show that there are a large number of mixed infections, and, as we want to further explore the pathogens of mixed infections, the metagenomics were used to detect the unknown pathogens.

## 2. Materials and Methods

### 2.1. Samples and RT-PCR Assays

Sixty-three calf diarrhea samples from Shijiazhuang, Heibei Province, manifesting as weakness, fever, excretion of watery feces, and accompanied by a foul smell, were subjected to RNA extraction using HiPure Total RNA Mini Kit (AnGen, Guangzhou, China). The total RNA was further reverse transcribed into cDNA using a Vazyme Reverse Transcription Kit (Vazyme, Nanjing, China). PCR amplification was performed using 2× Taq Master Mix (Vazyme, Nanjing, China) with the following primers: For BVDV: sense, 5′-AGCGGGGATAAGGTTGGAAA-3′; antisense, 5′-ACCTGCAGCCCCTTTTCTAT-3′. For BCoV: sense, 5′-GCTACCAATTATTTTGCTTGGC-3′; antisense, 5′-ATGGAGAGGGCACA GACTTATC-3′. For BoAstV: sense, 5′-GAYTGGACBCGHTWTGATGG-3′; antisense, 5′-K YTTRACCCACATNCCAA-3′. For BRoV: sense, 5′-ACCACCAAATATGACACCAGC-3′; antisense, 5′-CATGCTTCTAATGGAAGCCAC-3′ (Genscript, Nanjing, China). The above four pairs of primers were designed with reference to sequences GenBank accession no. KF501393, LC494178, LC047787, and MZ540977, respectively, using the Primer Premier 5.0 software package. The PCR reactions were performed with 2 µL of cDNA templates added to 18 µL of reaction mixture, containing final concentrations of 0.5 µM of each primer. The cDNA was amplified by 35 cycles of denaturation at 95 °C for 30 s, annealing at 55 °C for 35 s, and extension at 72 °C at 1 kb/min, and finally extended at 72 °C for 10 min.

### 2.2. Next Generation Sequencing (NGS)

One RNA sample positive for both BCoV and BoAstV was selected and sent to Shanghai Tanpu Biotechnology Co., Ltd. (Shanghai, China) for next-generation sequencing to obtain the sequences of these two viruses and to explore whether there were other viruses that may cause calf diarrhea. We used Illumina sequencing technology to complete the genome amplification and in-depth sequencing of the virus strain. Subsequent DNase treatment and cleanup was followed by second-strand synthesis before library preparation using Nextera XT reagents and sequencing on the NovaSeq 6000 (Illumina). Read quality trimming was performed using the Skewer, with an additional trimming filter for unreliable sequences after a user-specified quality score. Host read subtraction by read-mapping was performed with the BWASW program against ribosomal RNAs (16, 18, 23, 28, 5S, and internal transcribed spacers rRNA were retrieved from https://www.ncbi.nlm.nih.gov/, accessed on 15 March 2021), bacterial genome sequences, and the latest host organism genome sequences. We then used SPAdes and MEGAHIT software to de novo assemble the reads obtained after removal of the above-mentioned contamination sequence. The de novo assembly followed the A5-miseq pipeline. The final scaffolds were subjected to bwasw read mapping and a mega blast homology search against the NCBI NT database.

### 2.3. Completing the Sequence at Both Ends of the Genome

The sequences of the spliced BoAstV and BNoV were compared at the NCBI website (https://www.ncbi.nlm.nih.gov/, accessed on 25 April 2021), which showed that they had the highest sequence similarity to LC047787 and MN480761, respectively. Therefore, they were used as templates to design primers to amplify both ends of the whole genome of the virus. For BoAstV-5′: sense, 5′-CCAAAACAGGTCGGATTGAA-3′; antisense, 5′-TAGCTATTAAGACGACAGCT-3′. For BoAstV-3′: sense, 5′-GCATTAAGTTTAAGGCAGGC-3′; antisense, 5′- CCCCTTCACCTATGCTAATCA-3′. For BNoV-5′: sense, 5′-GTGAATGAAGACTTTGACGA-3′; antisense, 5′-AGGCCACGCGCCACCACGCT-3′. For BNoV -3′: sense, 5′-TGGAGTTGCAGGCTCGCTCT-3′; antisense, 5′-AACAATGCTGAATAGGAGGC-3′. The BKoV and BCoV-S sequences were directly spliced by Illumina sequencing without amplification at both ends by RT-PCR methods.

### 2.4. Phylogenetic and Genome Analysis

We used the ORF Finder (https://www.ncbi.nlm.nih.gov/, accessed on 12 May 2021) to predict open reading frames (ORFs) and their derived amino acid sequences. This method uses Clustal W software MEGA7.0 until genomic nucleotide sequences are aligned. Using the neighbor-joining method (neighbor-joining method, NJ), a phylogenetic tree was constructed, and an exhibit values (on Bootstrap) test was set to repeat 1000 times. At the same time, MegAlign software in the DNAstar software package was used to analyze the homology between these nucleotide sequences.

## 3. Results

### 3.1. Identification of BCoV and BoAstV Infection in Feces Samples by RT-PCR

The results showed that, in 48 samples out of 63 samples, at least one pathogen was detected, and the BCoV positive detection rate was the highest at 48.39%, followed by BRoV (26.98%), BoAstV (25.81%), and BVDV (19.05%). There are six types of mixed infections, BVDV + BCoV, BVDV + BoAstV, BCoV + BRoV, BCoV + BoAstV, BVDV + BCoV + BoAstV, BCoV + BRoV + BoAstV, and the infection rate is as high as 46.03% (Table 1). In addition, we detected two different BCoV sequences. BCoV and BoAstV were detected in the mixed infection sample numbered 210,041, therefore the high-quality total RNA was subsequently used for the following deep sequencing.

### 3.2. Viral Metagenomics

We extracted RNA from stool samples that tested positive for BoAstV and BCoV pathogens and then processed them for viral metagenomics using the Illumina HiSeq platform. The largest portion of the reads were assigned to the families Picornaviridae (*n* = 2992), Astroviridae (*n* = 819), and Caliciviridae (*n* = 187). We then generated complete or partial genome sequences for a subset of these viruses (Table 2).

### 3.3. Discovery and Analysis of BNoV

**Figure 1:**
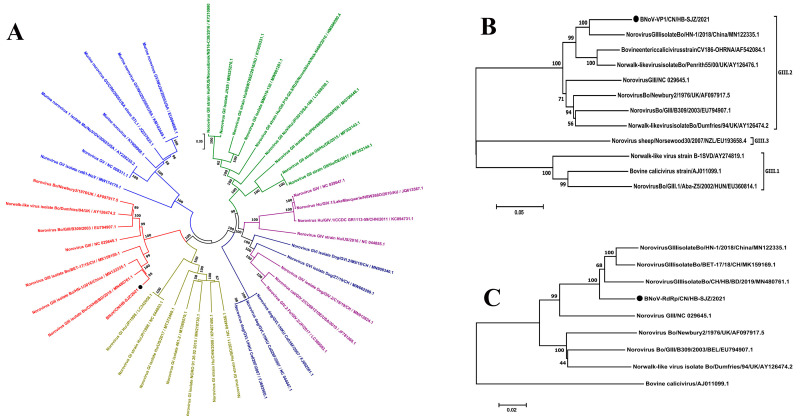
Phylogenetic analysis of BNoV/CN/HB-SJZ/2021: (**A**) Neighbor-joining unrooted phylogenetic trees based on the nucleotide sequences of the complete genomes of BNoV/CN/HB-SJZ/2021 and other genotypes. (**B**) Neighbor-joining unrooted phylogenetic trees based on the VP1 gene sequences of BNoV/CN/HB-SJZ/2021 and other GIII subtypes. (**C**) Neighbor-joining unrooted phylogenetic trees based on the RdRp gene sequences of BNoV/CN/HB-SJZ/2021 and other GIII subtypes. The bar represents a genetic distance. Numbers at nodes indicate bootstrap percentages obtained after 1000 bootstrap replicates. Our strain, BNoV/CN/HB-SJZ/2021, is marked with a black circle (●).

BNoV/CN/HB-SJZ/2021 genomic RNA consists of 7316 nucleotides. The BNoV genome contains three sequential ORFs starting from the 22 nt: ORF1, 5055 (22–5076) nt; ORF2, 1569 (5063–6631) nt; ORF3, 849 (6423–7271) nt, all of which encode a nonstructural polyprotein and structural VP1 and VP2 proteins, respectively. The G + C content of our strain genome was 57.50%, similar to that of the B309 (56.96%) and Newbury2 (56.71%) strains. Homology analysis shows that BNoV/CN/HB-SJZ/2021 and the NoravusGIII virus (MN480761) have 93.4% nucleotide characteristics (Table 3). Phylogenetic tree analysis shows the closest relationship with MN480761 (Figure 1a), as well as genetic evolution analysis, revealing that the VP1 protein is located in the same branch as the GIII.2 subtype (Figure 1b). Further analysis of the genetic evolutionary tree shows that the RdRp region of the virus also has the closest genetic relationship with MN480761 (Figure 1c).

### 3.4. BoAstV Acquisition and Analysis

**Figure 2:**
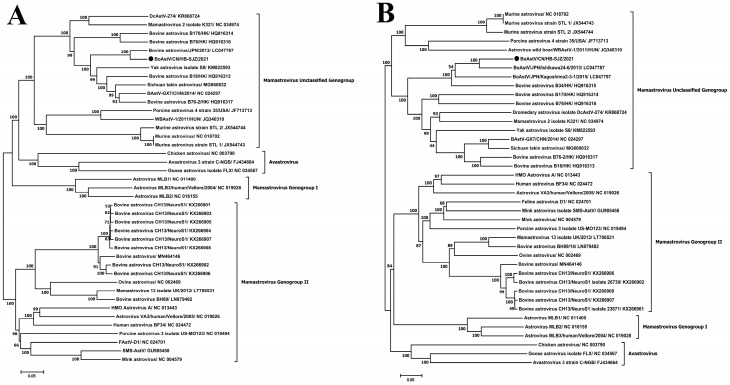
Phylogenetic analysis of BoAstV/CN/HB-SJZ/2021: (**A**) Neighbor-joining unrooted phylogenetic trees based on the nucleotide sequences of the full-length sequences of BoAstV/CN/HB-SJZ/2021 and different genogroups. (**B**) Neighbor-joining unrooted phylogenetic trees based on the ORF2 sequences of BoAstV/CN/HB-SJZ/2021 and different genogroups. The bar represents a genetic distance. Numbers at nodes indicate bootstrap percentages obtained after 1000 bootstrap replicates. Our strain, BoAstV/CN/HB-SJZ/2021, is marked with a black circle (●).

A complete genome of BoAstV/CN/HB-SJZ/2021 was sequenced and assembled. The BoAstV/CN/HB-SJZ/2021 genome was identical to the genome sequence assembled from metagenomics data. The size of the genome was 6324 bases and the G + C content was 52.25% (Table 2). The genome organization of BoAstV/CN/HB-SJZ/2021 was consistent with those of other BoAstV and deer astroviruses (deer AstVs), consisting of a 5′-UTR, three ORFs (ORF1a, ORF1b, and ORF2), and a 3′-UTR. The 5′-UTR and 3′-UTR consisted of 86 bases and 73 bases, respectively. The ORF2 of BoAstV/CN/HB-SJZ/2021 was predicted to encode a capsid protein of 767 amino acids. BoAstV/CN/HB-SJZ/2021 was most closely related to BoAstV/JPN/2013; it shared 86.0% nucleotide identity on the capsid protein (ORF2) region and was not so closely related to other Astrovirus species, including BoAstV B76-2/HK, BoAstV B18/HK, and Astrovirus Roe deer/SLO/D12-14, the three of which share 57.2%, 57.1%, and 56.5% nucleotide identities, respectively (Table 4) (Figure 2). As for genogroup, BoAstV/CN/HB-SJZ/2021 is not clustered with either genogroup I or II. Instead, it is closely related to a few unclassified astroviruses, including a few BoAstV, yak astrovirus, and dromedary astroviruses, suggesting that BoAstV/CN/HB-SJZ/2021 may potentially constitute a new genogroup in the genus Mamastrovirus (Figure 2a).

### 3.5. Discovery and Analysis of BKoV

**Figure 3:**
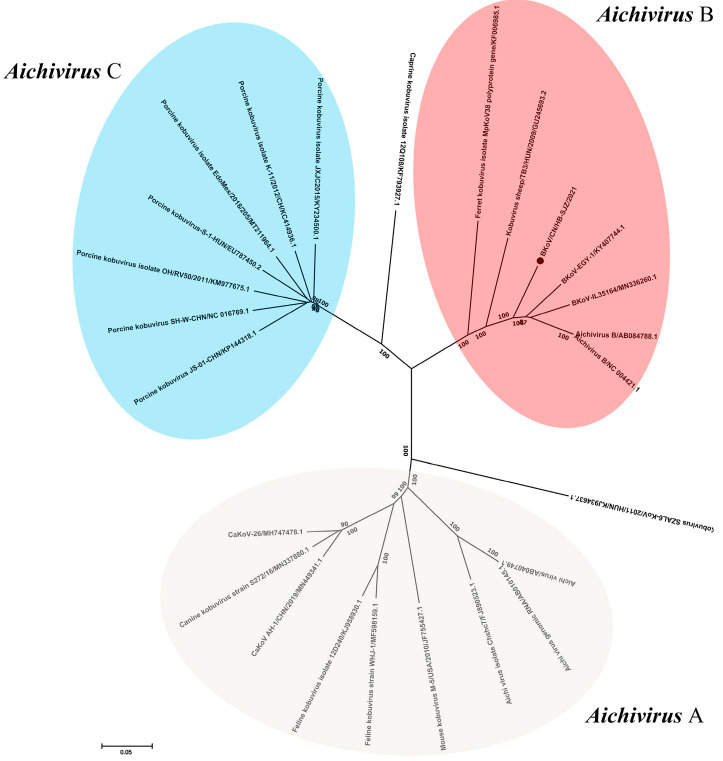
Phylogenetic analysis of BKoV/CN/HB-SJZ/2021: Neighbor-joining unrooted phylogenetic trees based on complete genomic sequences of BKoV/CN/HB-SJZ/2021 and other genus aichiviruses. The bar represents a genetic distance. Numbers at nodes indicate bootstrap percentages obtained after 1000 bootstrap replicates. Our strain, BKoV/CN/HB-SJZ/2021, is marked with a black circle (●).

The BKoV we obtained by sequencing contained 8376 base genomes. The BKoV/CN/HB-SJZ/2021 genome contained a large ORF, which encoded potential polyprotein precursors of 2568 amino acids. Reads were assembled into a complete genome contig that shares 88.4% nucleotide identity with the BKoV reference genome (MN336260), and genetic evolution analysis shows that the BKoV/CN/HB-SJZ/2021 belongs to the Aichi virus B genus (Figure 3). The 2C region of BKoV is relatively conserved, with nucleotide similarity reaching 62.7–89.2%. This region contains a highly conserved amino acid motif, GXXGXGKT, which is also recognized as the nucleic acid binding region of microRNA virus helicase. Therefore, 2C protein may be related to virus replication and nucleic acid uncoiling. There are three highly conserved amino acid motifs in the 3D region which encode the virus RNA polymerase. The nucleotide sequence similarity of the 3D region of the crest virus is the highest at 66.7–93.0% (Table 5).

### 3.6. Analysis of the Coronavirus Genome

**Figure 4:**
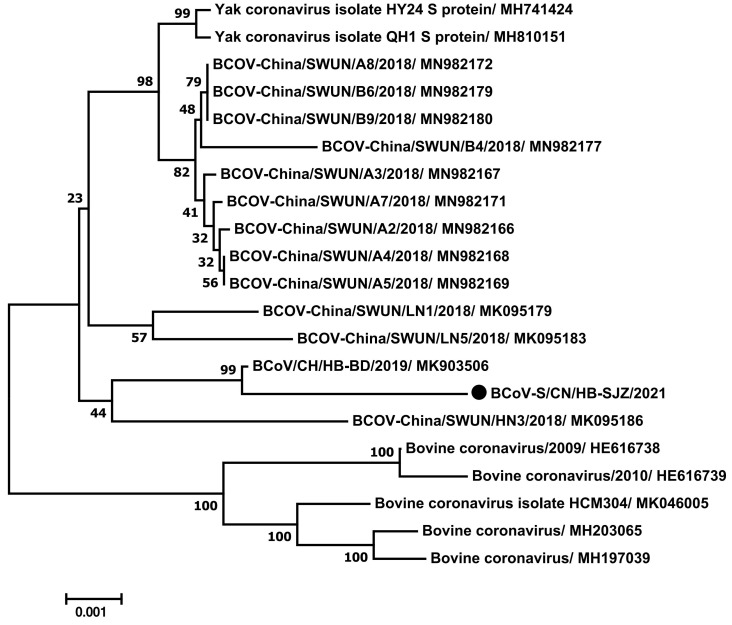
Phylogenetic analysis of BCoV/CN/HB-SJZ/2021: Neighbor-joining unrooted phylogenetic trees based on the S gene sequences of BCoV/CN/HB-SJZ/2021 and other BCoV. The bar represented a genetic distance. Numbers at nodes indicate bootstrap percentages obtained after 1000 bootstrap replicates. Our strain, BKoV/CN/HB-SJZ/2021, is marked with a black circle (●).

After BLAST alignment, we assembled the complete S gene sequence of BCoV through sequencing, and a sequence of 4092 nt length was obtained, encoding 1363 amino acid. The BCoV S genes from different parts of the world were selected as reference sequences. The complete phylogenetic tree of the S gene showed that, compared with the isolates from Vietnam and Cuba, the isolates formed a clear clade. In addition, BCoV-S/CN/HB-SJZ/2021 sequences closely related to BCoV/CH/HB-BD/2019 (MK903506) (Figure 4), which encodes the S protein, were identified (99.6%). It shared a anucleotide sequence higher than 99.1% with the Chinese strain, 98.4% similarity with the Vietnamese strain, and only 93.7% homology with the human coronavirus (Table 6). Sequence alignment shows that there are 17 base mutations with MK903506, and 6 mutations in encoded amino acids.

## 4. Discussion

Viral gastroenteritis remains as an important cause of morbidity and mortality in neonatal calves [8]. A considerable number of viruses in the gastrointestinal tract of calves are yet to be identified. Currently, the use of next-generation sequencing technologies facilitates virus detection and description. In this study, the metagenomics analysis revealed that four different viruses, BCoV, BoAstV, BKoV, and BNoV, were present in feces from calf diarrhea, and the distribution of these viruses was each associated with calf diarrhea. Additionally, a level of co-infection was detected, and co-infection or a specific combination of viruses is more likely to result in calf diarrhea, and requires the analysis of a larger number of cases and controls. A recent study has shown the presence of five viruses, including BToV, BCoV, BRoV, BVDV, and small round structured viruses (SRSV) in the feces of dairy calves, with a rate of co-infection of 14% in diarrheic calves, suggesting that mixed infections are common in calf populations [17].

The astroviruses reported here are commonly associated with gastrointestinal disease, particularly in immunodeficient infants [18,19], and they are generally detected in fecal samples [20,21]. Moreover, astrovirus can also been detected in the brains of humans [22] and animals [23], including cows, showing with neurological symptoms [24,25]; this indicates that the host’s range can extend beyond enteric tissues. However, the role of BoAstV as a causative agent of diarrhea in cattle is controversial and has not been extensively studied. Based on genomic sequences, ORF2 has greater variability among the three ORFs, and the characteristics of the ORF2 gene are important bases for AstV typing [26,27]. Analysis of the genetic evolution of the virus showed that, compared with other regions of the genome, the ORF2 region had the lowest similarity to other strains, which may suggest that a new serotype/genotype may appear. It is noteworthy that neonatal calf diarrhea is generally of a multifactorial origin; our study not only established an association between the presence of the virus and disease, but also determined the genetic diversity of BoAstV in herds from China.

BCoV has been associated with enteric and respiratory disease in cattle, including neonatal calf diarrhea (NCD) [28], winter dysentery (WD) [29], and respiratory tract illness [30]. All BCoV isolates identified so far that are shed in feces and nasal secretions belong to one serotype/genotype based on virus cross-neutralization and genotyping analyses, regardless of clinical origin [31]. However, there is no specific or effective method with which to prevent or control BCoV infection in China. In other countries, inactivated vaccines or attenuated vaccines are primarily used to immunize pregnant cows so that calves can ingest colostrum to improve passive immunity, or so that newborn calves can be used to stimulate active immunity [32]. However, a commercial BCoV vaccine was not available in China. S protein is a larger membrane glycoprotein that contains two hydrophobic regions and two subunits, S1 and S2, that are formed by cleavage at the 768 and 769 amino acids [33], which are mainly responsible for cell adhesion, blood coagulation, membrane fusion, and the induction of neutralizing antibodies. S1 is responsible for the recognition and binding of viruses and host cells, while S2 is related to the fusion between virus cells. The sequence comparison between BCoV-S/CN/HB-SJZ/2021 and its nearest strain, BCoV/CH/HB-BD/2019 (MK903506), showed that there are six amino acid mutation sites, of which four amino acid mutation sites are in the S1 subunit and two are in the S2 subunit. This indicates that most of the mutations are in the S1 region, and we can conduct in-depth research on this identification and combination area in order to provide more data for vaccine development and drug design.

To date, the pathogenesis of BNoV has been poorly understood, and the prevalence of BNoV in cattle has not been well established. In our study, BNoV was identified as the co-infection pathogen from diarrheic samples, along with other pathogens. Therefore, the true role of BNoV as a primary pathogen or as a form of co-infection remains unclear. Based on the phylogenetic relationships inferred from the VP1 sequences, noroviruses have been divided into six genogroups (GI to GVI), and BNoV is classified as GIII. The strain identified in our study was BNoV-GIII 2. Considering the previously reported GIII.1 strains identified in China [34,35]. It can be inferred that both genotypes GIII.1 and GIII.2 related sequences that have been found to circulate in Chinese dairy calves. Due to the limited epidemiological data on BNoV infections in China, the dominant strains cannot be accurately confirmed. Moreover, while different genotypes co-exist, ways of preventing diarrhea caused by BNoV are unclear. The results of our study will facilitate further research on the evolution and molecular pathogenesis of BNoV.

In 2008, BKoV was isolated from fecal samples of cattle with diarrhea, and it was concluded that BKoV played a role in the pathogenesis of enteritis in calves. However, the role of BKoV infection in calf diarrhea still needs to be clarified because of the presence of this virus in clinically normal animals [5]. In this study, through homology comparison, we found that the P1 gene showed as highly variable, while the 3D gene was highly conserved. Many early studies have shown that the BKoV-3D gene is mainly used to distinguish BKoV from other kobuviruses in phylogeny, rather than to differentiate BKoV into different lineages [36,37]. Therefore, the 3D gene may be used for designing specific primers for BKoV detection.

In summary, this study provided important information on at least four viral pathogens detected in diarrheic samples. These results will certainly contribute to the understanding of the evolution and pathology of BCoV, BoAstV, BKoV, and BNoV in cattle. Further epidemiology studies will reveal whether these viruses can also be found in other diarrheic samples, as well as their pathogenic potentials. Next-generation sequencing, as a new method, can discover more unknown viruses in samples, better understand the causes of calf diarrhea, and provide powerful technical support for the prevention and control of diarrheal diseases.

## Figures and Tables

**Table 1:** Detection of viruses in calves showing diarrhea.

	Total Infection				Single Infection					Mixed Infection						
	a *	b *	c *	d *	a	b	c	d	Total	a + b	a + d	b + c	b + d	a + b + d	b + c + d	Total
Number of Positive Detections	12	30	17	16	2	10	5	2	19	7	3	11	1	6	1	29
Positive Infection Rate (%)	19.05	48.39	26.98	25.81	3.17	15.87	7.94	3.17	30.16	11.11	4.76	17.46	1.59	9.52	1.59	46.03

* a: BVDV; b: BCoV; c: BRoV; d: BoAstV.

**Table 2:** Genomic information of the four viruses.

Virus	Species	GenBank Accession No.	Base Size (nt)	Amino Acid Size (aa)	GC (%)
BNoV/CN/HB-SJZ/2021	Norovirus GIII.2	MZ573179	7316 (7276) *	2488	57.50
BoAstV/CN/HB-SJZ/2021	Mamastrovirus Unclassified Genogroup	MZ603733	6324 (6284) *	1965	52.25
BKoV/CN/HB-SJZ/2021	Aichivirus B	MZ603734	8376	2568	55.40
BCoV-S/CN/HB-SJZ/2021	Embecovirus	MZ603735	4092	1363	35.78

* The sequence exclude the primer binding regions.

**Table 3:** The nucleotide identities of BNoV/CN/HB-SJZ/2021 compared to different norovirus isolates.

Nucleotide Identity (%)						
	Norovirus GI (MT372469) Human	Norovirus GII (KY210980) Human	Norovirus GIII (MN480761) Cattle	Norovirus GIV (NC029647) Human	Norovirus GV (MW174170) Norway Rat	Norovirus GVI (MN908340) Dog
Complete genome	58.3	51.6	93.4	53.3	50.8	53.6
p48	53.1	44.9	92.8	46.8	45.3	47.5
NTPase	64.7	55.1	94.3	56.1	53.2	58.3
p22	46.4	46.3	90.0	46.6	43.9	44.7
VPg	55.1	53.3	92.1	55.7	52.7	58.6
Pro	59.1	63.7	96.9	61.5	59.6	63.7
RdRp	66.5	59.2	94.3	60.9	58.5	63.0
ORF2-VP1	56.2	49.2	93.3	51.1	50.2	52.3
ORF3-VP2	41.8	42.7	92.2	43.9	42.0	41.7

**Table 4:** The nucleotide identities of BoAstV/CN/HB-SJZ/2021 compared to different astrovirus isolates.

Nucleotide Identity (%)							
	Mamastrovirus Undassified Genogroup					Mamastrovirus Genogroup I	Mamastrovirus Genogroup II
	Bovine (LC047787)	Bovine (HQ916317)	Roe Deer (MN150125)	Bovine (HQ916313)	Porcine (JF713713)	Human (NC_011400)	Ovine (NC_002469)
Complete genome	88.4	77.0	72.3	81.4	77.3	64.0	56.4
ORF1ab	95.8	87.7	80.9	73.3	52.9	44.9	46.0
ORF2	86.0	57.2	56.5	57.1	46.5	41.3	38.9

**Table 5:** The nucleotide identities of BKoV/CN/HB-SJZ/2021 compared to different kobuvirus isolates.

Nucleotide Identity (%)										
	Aichivirus A				Aichivirus B			Aichivirus C	European Roller Kobuvirus	Caprine Kobuvirus
	Human (AB010145)	Canine (MH747478)	Feline (KJ958930)	Mouse (JF755427)	Bovine (MN336260)	Sheep (GU245693)	Ferret (KF006985)	Porcine (EU787450)	Coracias Garrulus (KJ934637)	Caprine (KF793927)
Complete genome	58.4	58.7	58.5	58.4	88.4	81.4	77.3	64.0	56.4	64.3
P1	57.5	58.0	58.4	57.3	84.0	75.8	76.0	63.8	57.1	62.3
2C	68.1	68.0	65.9	65.6	89.2	85.9	82.4	72.0	62.7	70.5
3D	69.4	69.6	71.2	69.8	93.0	90.3	85.4	73.9	66.7	75.9

**Table 6:** The nucleotide identities of BCoV/CN/HB-SJZ/2021 compared to different coronavirus isolates.

Organism	GenBank Accession No.	Host	Country	Year	Nucleotide Identity (%)
BCoV	MK903506	Cattle	China	2019	99.6
	MK095179	Cattle	China	2018	99.1
	MH197039	Calf	Vietnam	2017	98.4
	MH203065	Calf	Vietnam	2017	98.4
Canine respiratory coronavirus	AY150272	Canine	The United Kingdom	2002	95.8
	AB242262	Canine	Japan	2005	96.0
	EU983107	Canine	South Korea	2008	95.8
Human coronavirus OC43	AY903454	Human	Belgium	2003	93.7
	KF963234	Human	France	2003	93.7
	AY903457	Human	Belgium	2003	93.7

## Data Availability

All data used and presented in this study is either available in public repositories as described in the Methods section or is made available in NCBI database.
